# Co-contamination of metal (loid)s and antibiotics induced the enrichment of antibiotic resistance genes in the coastal industrial basin

**DOI:** 10.3389/fmicb.2026.1777820

**Published:** 2026-04-01

**Authors:** Changpeng Sang, Wenyue Chen, Enrui Li, Meihua Lian, Zhiheng Li, Xiaojun Li

**Affiliations:** 1 Key Laboratory of Industrial Ecology and Environmental Engineering (Ministry of Education, China), School of Environmental Science and Technology, Dalian University of Technology, Dalian, China; 2 Key Laboratory of Pollution Ecology and Environmental Engineering, Institute of Applied Ecology, Chinese Academy of Sciences, Shenyang, Liaoning, China; 3 Key Laboratory of Wastewater Treatment Technology of Liaoning Province, Shenyang Ligong University, Shenyang, China; 4 School of Environmental Science and Engineering, Zhejiang Gongshang University, Hangzhou, Zhejiang, China

**Keywords:** antibiotics, ARGS, co-resistance, heavy metal (loid)s, MGEs

## Abstract

Sediment is the sink of antibiotic resistance genes (ARGs) in aquatic systems. The pollution of ARGs in sediments is becoming severe and complicated. However, the relationships between heavy metals/antibiotics with ARGs in sediments are unclear. Thus, we collected river sediment samples to investigate the effects of 18 antibiotics/8 heavy metal (loid)s on ARGs' contamination. Results showed that tetracycline, lipopeptide and sulfonamide were 100% detection, where tetracyclines were the most abundant, ranging from 44 ng/g to 408 ng/g dw. Meanwhile, arsenic, zinc, and cadmium were the most seriously contaminated with the concentrations of 4–812 mg/kg, 155–6158 mg/kg, 0.1–23 mg/kg, respectively. The abundance of ARGs (*tetA, sul1, sul2*, and *qnrA*) were not only closely with antibiotics, but also significantly positively correlated with heavy metals. What's more, the heavy metals had significant positive correlations with MGEs such as plasmids, integrons, and efflux pumps, suggesting that heavy metals could potentially drive the propagation of ARGs in sediments. The rand-forest model showed the heavy metal (loid)s contributed up to 70% to the ARGs pollution in sediments. This study extends our understanding of the correlation between ARGs and heavy metals/antibiotics and emphasizes the importance of considering co-existing heavy metals in the ARG pollution in coastal watersheds.

## 1 Introduction

Antibiotic resistance genes (ARGs) are a new type of contaminants in recent years, and the risk of horizontal gene transfer has been identified by the World Health Organization as a major challenge in health security in the Twenty-first century. ARGs contamination is mainly caused by antibiotics and antibiotic residues. On the one hand, they can be used to treat human diseases, and on the other hand, they can act on plants and animals as feed and fertilizer (Zhu et al., 2013). Sewage discharged from human activities contains a large number of ARGs, which will enter the soil and water environment through water reuse and atmospheric deposition. Soil and sediments are equivalent to a large-scale transit station for the transformation and expansion of resistance genes. A large number of antibiotic preparations are used in animal husbandry and agricultural practices, which results in the direct discharge of a large number of ARGs into the environment. In China, the current high residual antibiotics are mainly tetracycline, quinolone and sulfonamide antibiotics, but there are regional differences. This leads to serious contamination of the environment with ARGs, and resistance genes can be transferred between human organs through horizontal gene transfer, or can enter the human or animal body through the food chain, posing a potential health threat, and in severe cases rendering antibiotic treatment ineffective.

In the face of ARGs contamination, we had to control the use of antibiotics and reduce the emission. But it has been found to be sometimes ineffective in controlling the spread of ARGs in sediments, suggesting that there are other factors in the environment that drive the spread of ARGs, such as heavy metal (loid)s (Di Cesare et al., 2016). Rasmussen's study of Danish harbor waters found that strains isolated from heavy metal-polluted waters contained more resistant plasmids than strains isolated from non-heavy metal-polluted waters (Rasmussen and Sorensen, 1998). Like antibiotics, heavy metals are also important selection pressures for the spread of ARGs in the environment. Heavy metal (loid)s have similar resistance-generating mechanisms as antibiotics, and can exert selective pressures on ARGs in three ways: co-resistance, cross-resistance, and co-regulation (Wright et al., 2006). Unlike antibiotics, metal (loid)s is not easily degraded, and can exert long-term selective pressures. Some results have shown that when the Cu content in soil reaches a certain value, it affects the abundance, community structure and function of soil microorganisms, leading to a decrease in soil quality and disruption of ecological structure (Chen et al., 2018; Lin et al., 2019).

In addition, ARGs in the environment can realize horizontal gene transfer (HGT) between bacteria of the same or even different species with the help of MGEs, such as plasmids, integrons, transposons, etc., thus accelerating the spread and propagation of ARGs, which is potentially harmful to the environment and human health. However, current research is mostly focused on soil resistance, it is still unclear whether there are similar patterns in nearshore sediments. Klasen showed that silver and sulfonamide resistance genes are located on the same replicon and suggested a mediating role of heavy metal (loid)s in the production of ARGs (Klasen, 2000). However, the extent to which metal (loid)s content affects the distribution of ARGs is not known.

In this study, the Jinzhou Bay area, where the metal smelting activities are often carried out, is selected as the research object. Jinzhou Bay is located on the western coast of Liaoning Province, which is rich in mineral resources, and at the same time, it is the nearest sea port in the northeast region. With the development of society, the dense population living and various industrial and agricultural activities have had a significant impact on Jinzhou Bay and the nearby estuaries. The objectives of this study were: (1) to investigate the distribution of ARGs, antibiotics and heavy metal (loid)s in all the samples, (2) to explore the correlation between heavy metal (loid)s, antibiotics and ARGs, and (3) to study the effects of antibiotics and heavy metal (loid)s on ARGs in sediments. The study helps us to re-conceptualize the effects of heavy metals on antibiotic resistance genes in compound-contaminated sediment environments.

## 2 Materials and methods

### 2.1 Sampling locations and sample collection

**Figure 1:**
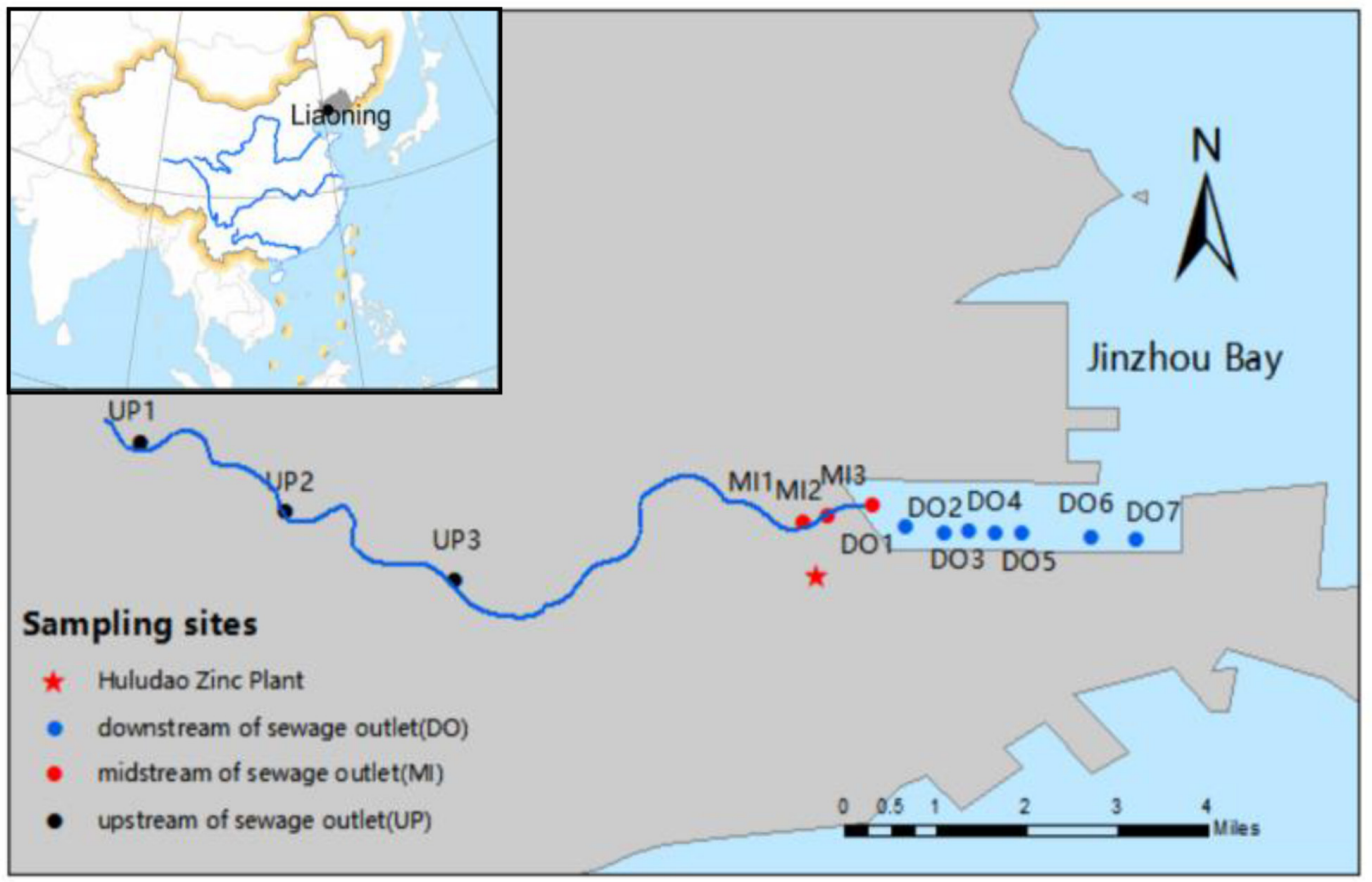
Map of the study area and sampling sites.

In June 2019, a total of 13 sediment sampling sites were established along the Jinzhou Bay coastline, distributed upstream, midstream, and downstream of wastewater outlets as follows (Figure 1): upstream of outlets (UP): UP1, UP2, UP3; midstream of outlets (MI): MI1, MI2, MI3; downstream of outlets (DO): DO1 through DO7. Surface sediment samples (0–5 cm depth) were collected at all sites using an ethanol-sterilized shovel. All samples were immediately transported to the laboratory and stored at −20 °C for subsequent chemical analysis and DNA extraction. Additionally, vertical profile sampling was conducted at two representative sites, DO1 and MI3. Sediment cores were collected at 5 cm intervals from the surface down to 60 cm depth using a handheld trowel. Samples from each depth interval were placed in sealed bags, wrapped in aluminum foil to protect them from light, and labeled with the sampling time, site identifier, and target analyses.

### 2.2 Heavy metal (loid)s analysis

Determination of the total concentrations of metal (loid)s in the sediment: Cd, Co, Pb, Mo, Ni, Cu, and Zn were measured by acid digestion using inductively coupled plasma mass spectrometry (ICP-MS, NexION300x, PerkinElmer, USA), with a detection limit of 1 ug/L. As was measured by mixed acid digestion using a hydride generation-atomic fluorescence spectrometer (AFS, AFS-2202E Haiguang, China), with a detection limit of 10 ug/L. In order to ensure the accuracy and reliability of the data, all reagents used in the entire analysis process were of superior purity, the experimental water was deionized water, and all glassware was soaked in 10 % nitric acid for >24 h. Quality assurance and quality control were carried out using blank, duplicate, and national standard samples (GBW07314, “Standard Reference Material for Component Analysis of Marine Sediments near the Coast,”) with a recovery range of 95 %−105%. Determination of the bioavailable concentrations of metal (loid)s in the sediment: Fe, Cd, Co, Pb, Mo, Ni, Cu, and Zn were measured using an improved BCR four-step extraction method (Tessier et al., 1979) to extract weak acid extractable, reducible, oxidizable, and residual metal elements successively. The weak acid extractable, reducible, and oxidizable states were considered as bioavailable concentration and were measured using ICP-MS.As was measured using the continuous extraction method as previously described (Wang et al., 2012), which successively extracted exchangeable, acid volatile sulfide/carbonate/manganese oxide/amorphous iron oxide coprecipitated, iron oxide coprecipitated, pyrite and organic matter-bound states, and residual state. The exchangeable and carbonate-bound states were considered as bioavailable concentration and were measured using a hydride generation-atomic fluorescence spectrometer (AFS, AFS-2202E Haiguang, China).

### 2.3 Analysis of antibiotics

The sediment samples were first freeze-dried for more than 48 h. Subsequently, 0.1 g of sludge was collected and 15 mL of organic extractant and 15 mL of water extractant were added. The organic extractant was a mixture of MeOH, ACN, and EtOAc (v: v: v = 1:1:2), and the aqueous extractant was a buffer solution of 0.2 mol/L NaH_2_PO_4_, pH 3.0, containing 0.001 mol/L Na_2_EDTA. The mixture of sediment and extractant was vortexed thoroughly for 5 min at room temperature and sonicated for 20 min. the mixture was centrifuged at 1,000 rpm for 5 min and the supernatant was collected. The residual solids were extracted more than twice with organic and aqueous extractants. After extraction, the supernatant was evaporated under vacuum at 50 °C for 10 min to remove organic solvents. After collecting the residue, all the liquid was collected into 100 mL glass vials and the glass vials were washed with 5% methanol. Subsequently, the collected liquid was evaporated to 0.1 mL at 40 °C with a gentle stream of pure nitrogen gas. The residual liquid was then diluted to about 0.4 mL with 50% methanol solution and transferred to a 1 mL volumetric flask. The tube was then rinsed three times with 50% methanol solution and all liquid was collected into the same 1 mL volumetric flask. The volume of 1 mL was accurately obtained by adding 50% methanol solution to the volumetric flask. The re-dissolved solution was then filtered through a 0.22 μm membrane into a 2 mL vial for on-line analysis. Pretreated sediment samples were analyzed using solid phase extraction (SPE) and triple quadrupole column liquid chromatography tandem mass spectrometry (HPLC-MS/MS, Thermo TSQ Quantitiva, USA) (Tian et al., 2022)to analyze antibiotic species and levels in the samples. We detected 18 antibiotics in the samples, which were AMO (amoxicillin), AMP (ampicillin), AZI (azithromycin), EYR (erythromycin), ROX (roxithromycin), CIP (ciprofloxacin), ENR (enrofloxacin), and NOR (norfloxacin), OFL (ofloxacin), CTC (chlortetracycline), OTC (oxytetracycline), TC (tetracycline), DTC (daptomycin), SDZ (sulfadiazine), SMR (sulfadiazine-methyl-pyrimethamine), SMT (sulfadimethyl-pyrimethamine), SMZ (sulfonamides) TMP (methicarbamol), which were classified into six major groups according to the different characteristics of the resistance for statistical analysis.

### 2.4 Analysis of ARGs and MGEs

DNA was extracted from the sediments using a DNA isolation kit (MoBio). DNA quality and concentration were then measured using a Nano Drop 2,000 spectrometer (Thermo Fisher Scientific). Data were collected from a total of 8 ARGs (*qnrA, qnrS, sul1, sul2, tetA*, and *tetM*).The quantification of selected genes was conducted under the Step One Plus Real-Time PCR Systems (ABI, USA) in three replicates (Aminov et al., 2001; Chen and Zhang, 2013; Luo et al., 2010). The primers of real-time PCR were the same as those in the PCR process. The real-time PCR system was carried out in triplicate with a final volume of 15 μL, which consisted of 7.5 μL of SYBR Premix Ex Taq™ (TaKaRa), 4.6 μL of double-distilled water, 0.3 mL of the 50 × ROX reference dye, 0.3 μL of the forward primer (10 mM), 0.3 μL of the reverse primer (10 mM) and 2 μL of the template DNA.

Six sediment samples were chosen for metagenomic sequencing on Illumina Hiseq 4,000 platform by Majorbio (Shanghai, China). Raw reads were quality filtered by Trimmomatic (Bolger et al., 2014), then assembled using Megahit (Li et al., 2015). For sequencing methods refer to our previous article (Zeng et al., 2023). Resistance mechanisms and movable genetic elements were selected for analysis in the test results of the macrogenome section.

The relative abundance of ARGs was calculated to normalize for variations in sequencing depth across samples. The raw read counts for each ARG, obtained by aligning reads to the curated ARG database (as described above), were normalized to copies per million (CPM) using the following formula:

Relative Abundance (CPM) = (Read count of a specific ARG/Total reads in the sample) × 1,000,000

This normalization was performed using the MetaWRAP Quant Bins module (Uritskiy et al., 2018), which was also applied to quantify gene abundance in the metagenomic analysis.

### 2.5 Data analysis

Distribution of sampling sites was done using ArcMap 10.2. Histograms and Pearson/Spearman correlation plots were plotted using OriginLab 2022 to analyze the content and concentration of antibiotics, heavy metals and the correlation between resistant genes and them. The random forest (RF) model was used to identify the importance of heavy metals and antibiotics on the distribution of ARGs. The response variables were bootstrap resampling to generate untrimmed decision trees (1,000 trees in this study). Then, the best split of each tree was assessed using randomly 1/3 of all variables. The OOB predictions were aggregated to calculate the mean square error (MSE). The importance of each variable predictor indicates the increase in the mean square error between the observed value and the OOB prediction (increase of MSE). These analyses were performed using the “Random Forest” R package.

## 3 Result and discussion

### 3.1 Status of heavy metal (loid)s and antibiotics in sediments

#### 3.1.1 Distribution of heavy metal (loid)s in sediments

**Figure 2:**
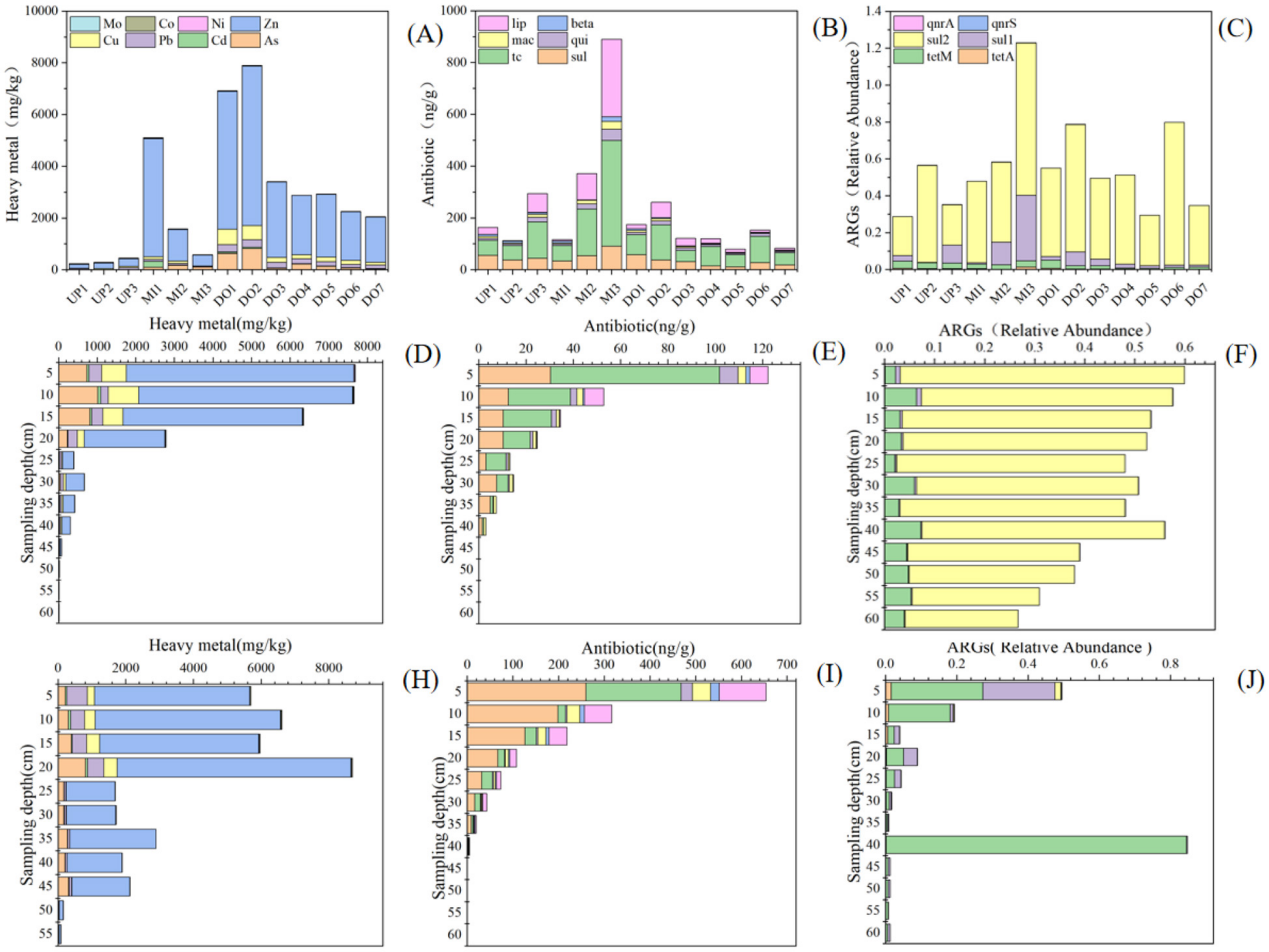
The contents of heavy metals and antibiotics, and the abundance of ARGs at different sampling sites. **(A–C)** was the surface sampling sites. **(D–F)** was the DO1 profile sites. **(H–J)** was the profile sites.

From the results of heavy metal content analysis, the heavy metal content in the middle reaches was significantly higher than that in the upper reaches, where levels were comparatively low. The pollution condition in the middle reaches of the outfall and the downstream area is very serious, with the content of Zn as high as 70%, the content of As reaches 20%, and the total amount of heavy metal can reach up to 22 g/kg (Figure 2A), leading to the serious pollution of heavy metal. The pollution condition of the profile had a similar pattern to the surface layer, and high heavy metal contents were still detected up to a depth of 20 cm (Figures 2D, H).

We noticed that high levels of ARGs were also detected at sites with high levels of heavy metals, and the patterns of heavy metal accumulation in the surface sediment and the two profile samples had similar patterns to those of ARGs. In previous studies, the interactions between bacterial communities and the environment in the sediments with different levels of contamination by metal elements in this area were also revealed. Comparison between the areas affected by sewage discharge and the relatively unpolluted areas revealed significant changes in the diversity, structure and composition of the bacterial communities, suggesting that the bacterial communities may have been affected by metal element pollution (Zeng et al., 2023).

#### 3.1.2 Distribution of antibiotics in sediments

According to the reports of Lyu et al. (2020), the detection rates of antibiotics in soil, surface water and coastal water in China were 100%, 98.0%, and 96.4%, respectively, with antibiotic contamination being the most serious in the Bohai Bay region. In addition to heavy metal contamination, the antibiotic contamination situation in the region was also very serious, but the antibiotics in different sediments were not the same. In our study, the highest antibiotic levels in the region were tetracyclines, followed by sulfonamides, lipopeptides, and quinolones. The antibiotic levels near the discharge outlet were more than three times higher than those in areas unaffected by point-source pollution, with significant enrichment on a regional scale and a sharp increase in tetracycline and lipopeptide antibiotics, with tetracycline antibiotics reaching a maximum of 409 ng/g, lipopeptide antibiotics reaching a maximum of 300 ng/g, sulfonamide antibiotics reaching a maximum of 90 ng/g, and quinolone antibiotics reaching a maximum of 42 ng/g (Figures 2B, E, I). Antibiotics had similar distribution patterns with heavy metals and ARGs, and pollution enrichment occurred at the same points. It may be due to the presence of antibiotics or resistant microorganisms in the discharged effluent, in addition, factors such as atmospheric deposition, animal husbandry, and facility-based agriculture might have the effect on the distribution of antibiotics.

### 3.2 Abundance of ARGs in sediments

The antibiotic resistance genes detected from the samples were mainly *tetA, tetM, sul1, sul2, qnrS*, and *qnrA*. This coincided with the common types of resistance genes in China in previous studies (Zhao et al., 2018). The relative abundance of ARGs in surface sediments varied from 0.195 to 1.23, and the average value could reach 0.53, which was comparable to the abundance of ARGs in soils of facility vegetable fields in some areas (Zhou et al., 2017). The midstream area of ARGs in the surface sediment had the highest content, followed by the downstream area, and the least content was in the upstream area. The two profiles in the midstream (MI3 site) and downstream (DO1 site) had a similar pattern of decreasing ARGs with depth, but the downstream (DO1 site) profile had a higher content of ARGs.

In the surface sediments, we found that ARGs were mainly dominated by *sul2* and *sul1*, because sulfonamide ARGs were more stable than tetracyclines, which may be related to the widespread use of sulfonamides in the treatment of pathogens, their high water solubility and persistence (Guo et al., 2014; Stoob et al., 2007). In contrast, the highest percentage of ARGs in the downstream (DO1 site) profile was *sul2* and *tetM*, and the highest percentage of ARGs in the midstream (MI3 point location) profile was *sul1* and *tetM*. In contrast, the downstream was more seriously contaminated with ARGs, and the gene abundance of 0.266 was still detected until the depth of 60 cm, which may be due to the fact that this point is located in the downstream of the wastewater outflow location, which was more affected by the enrichment of various pollutants (Figures 2C, F, J).

Sulfonamide antibiotics target dihydrogen phosphate synthase (DHPS) in the folate pathway, and resistance was generated by mutation of the DHPS gene or acquisition of another DHPS gene, *sul2* and *sul1* were two selectable DHPS (Ji et al., 2012); *tetM* encoded a cytoplasmic protein that confers a mechanism of tetracycline resistance on ribosomal protection, and the *tetA* gene was the most common tetracycline resistance gene in Gram-negative bacteria and encoded the tetracycline efflux pump *tet*A; *qnr* gene was a novel mechanism to cause quinolone drug resistance, mediated by plasmid which carries multi-drug resistance genes, leading to multi-drug resistance. The level of quinolone resistance induced by *qnrS* genes was directly correlated with their expression levels, and when the *qnrS* genes were present in high-expression environment, they may generate genetically stable clinical resistance in one step (Elena et al., 2020).

### 3.3 Correlation of antibiotics and heavy metals with the abundance of ARGs

**Figure 3:**
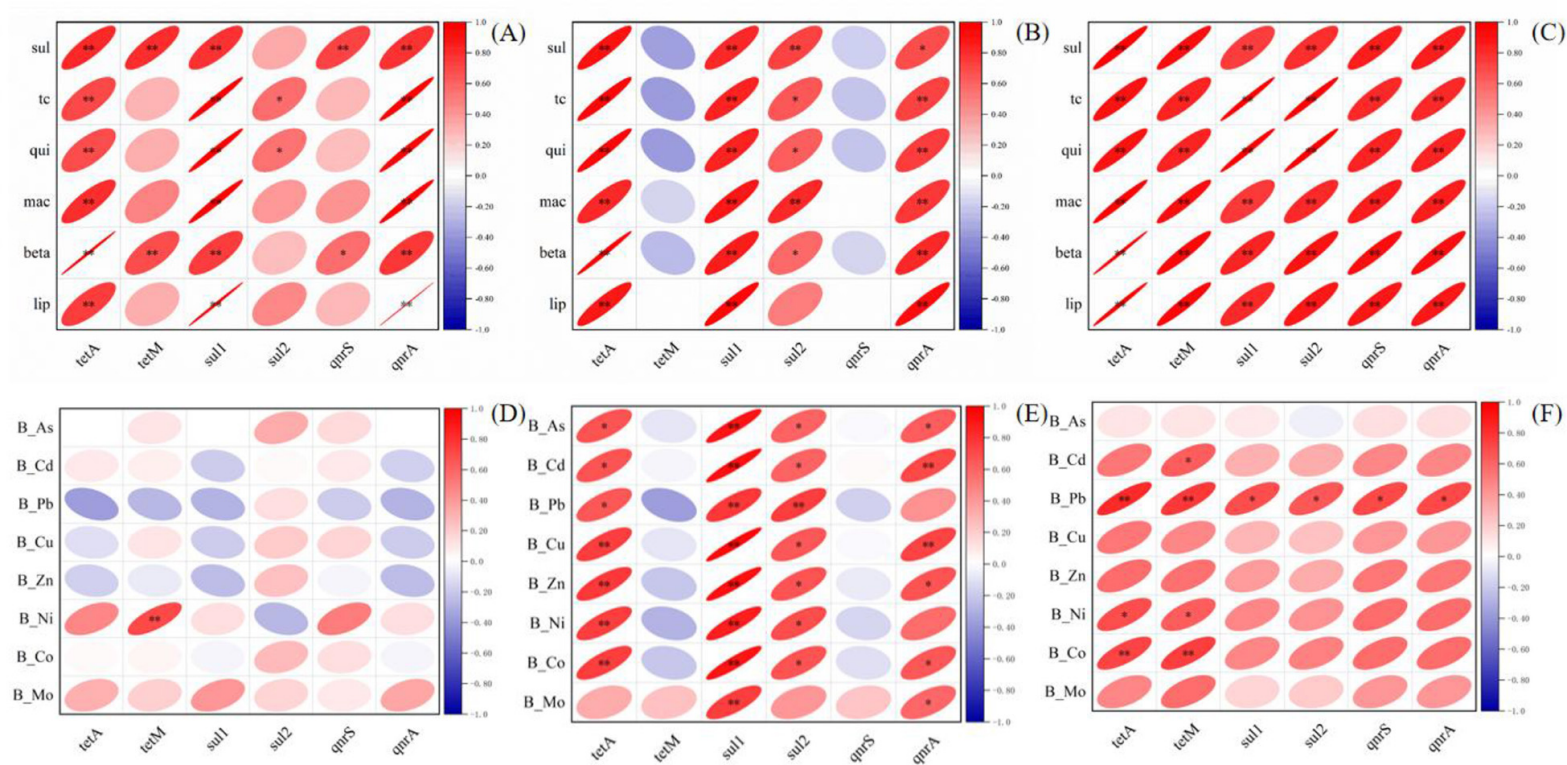
The effects of heavy metals and antibiotics on the ARGs. **(A, D)** was the surface sampling sites. **(B, E)** was the DO1 profile sites. **(C, F)** was the profile sites.

The correlation relationship between six major resistance genes and six different classes of antibiotics was analyzed, and it was found that *tetA, sul1*, and *qnrA* gene abundance were significantly and positively correlated with all types of antibiotics in the river surface sediment; while *tetM* was significantly and positively correlated with the content of sulfonamide and β-lactam antibiotics; *sul2* was positively associated with tetracycline and quinolone antibiotics; and *qnrS* was positively correlated with quinolones and β-lactams. In the downstream (DO1 site) profile, *tetA, sul1*, and *qnrA* genes were significantly and positively correlated with all types of antibiotics, similar to the pattern in surface sediments; whereas *tetM* and *qnrS* had no significant correlation with the content of all types of antibiotics; *sul2* had a positive correlation with all of the delipeptide antibiotics. In th midstream (MI3 site) profile, resistance genes had significant positive correlations with all types of antibiotics (Figures 3A–C). In this study, these weak correlations between antibiotics and their corresponding resistance genes may be due to different environmental fates as well as the transport mechanisms of resistance genes and antibiotics after release (Peak et al., 2007). Because surface sediments are susceptible to river migration and human activities, the patterns presented are not as pronounced as in the profile samples.

The modes of action of the six classes of antibiotics mentioned in this paper were presented in the figure (Supplementary Figure 1) (Alekshun and Levy, 2007; Sengupta et al., 2013; Sultan et al., 2018), and in addition to the selection that occurs after exposure to antibiotics, other factors may play a role in the observed inconsistency in the correlation between antibiotics and ARGs. For example, cross-selection may disrupt correlations if any of the six ARGs are located on a mobile genetic element, said mobile genetic element also containing a different class of resistance genes subjected to stronger selective pressure, such as heavy metals or another class of antibiotics (Wardwell et al., 2009). In the present study, the stronger correlation between ARGs and different types of antibiotics could be a result of this cross-selection.

The correlations between the six major resistance genes and nine different heavy metal (loid)s were analyzed using Pearson's correlation analysis, and it was found that the *tetA* and *tetM* genes were negatively correlated with the content of Mo, *tetM* was also positively correlated with the content of Ni, and the rest had no significant correlations in the surface sediments of the river (Figure 3D). The weak correlation between surface sediments and ARGs may be partly due to the fact that heavy metal genotoxicity is mainly associated with the dissolved fraction of the sediment rather than the particulate fraction of the sediment (Codina et al., 2000). In the downstream (DO1 site) profile, *tetA* and *sul2* genes were positively correlated with all heavy metal (loid)s except Mo; whereas *tetM* and *qnrS* had no significant correlation with the content of all types of heavy metals; *sul1* was significantly positively correlated with all types of heavy metal (loid)s; and *qnrA* was positively correlated with heavy metals except Pb and Ni (Figure 3E). In the midstream (MI3 site) profile, all types of resistance genes were positively correlated with Pb; Ni, Co, Fe, and Cd were positively correlated with *tetA* and *tetM* genes, and the rest were also weakly positively correlated (Figure 3F).

The relationship between heavy metal (loid)s and the abundance of resistance genes has been proved by many references, for example, Zhang et al. (2018) found that the concentration of Zn and Cu had a significant effect on the relative abundance of sul1 gene by analyzing the detection of heavy metals and the level of ARGs in agricultural soils. He et al. (2017) demonstrated that the exposure of Cu and Zn in fish ponds could promote the proliferation of ARGs, and that the proliferation of tetracyclines and sulfonamides The total abundance of ARGs increased by about 10% and 27%, respectively. It has also been shown (Seiler and Berendonk, 2012) that long-term soil exposure to Ni contamination resulted in elevated ARGs species and abundance, with the relative abundance of ARGs in tidal soils significantly increasing from 61–72 copies/g to 114–121 copies/g at a Ni concentration of 400 mg/L, and the relative abundance of ARGs in red soils similarly increasing from 45–64 copies/g to 116–126 copies/g, which may be due to the bioavailability of Ni and the moveable genetic elements effects. Knapp et al. (2011) investigated the relationship between heavy metals and ARGs in soil and showed that a variety of ARGs were positively correlated with the levels of Cu, while Cr, Ni, and Pb were also significantly correlated with specific genes. In the correlation analysis, antibiotics were better correlated with resistance genes, but after random forest analysis, it was found that for resistance genes, the heavy metal contribution was 20.67%−72.62% and antibiotic contribution was 27.38%−79.33%. This variability was attributed to differences among specific genes, with Cu, Co, and Mo exhibiting relatively significant influence. It indicated that heavy metal (loid)s also significantly affected the distribution of ARGs (Supplementary Table 4).

This study shifts the focus from total metal concentrations to heavy metal bioavailability. The speciation analysis reveals that bioavailable fractions—particularly weak acid-extractable and reducible forms—are the primary drivers of ARG proliferation. It revealed that the acid-extractable and reducible HMs were the active factors driving the spread of ARGs and MGEs. Notably, these fractions exert selective pressure on critical ARGs (e.g., sul1 and sul2) that rivals or exceeds that of co-existing antibiotics, with heavy metals contributing up to 72.6% to sul gene variation. This paradigm shifts from monitoring total pollutants to assessing bioavailable fractions provides new mechanistic insight into co-selection pressures in coastal ecosystems.

### 3.4 Correlation of antibiotics and heavy metals with the abundance of MGEs

**Figure 4:**
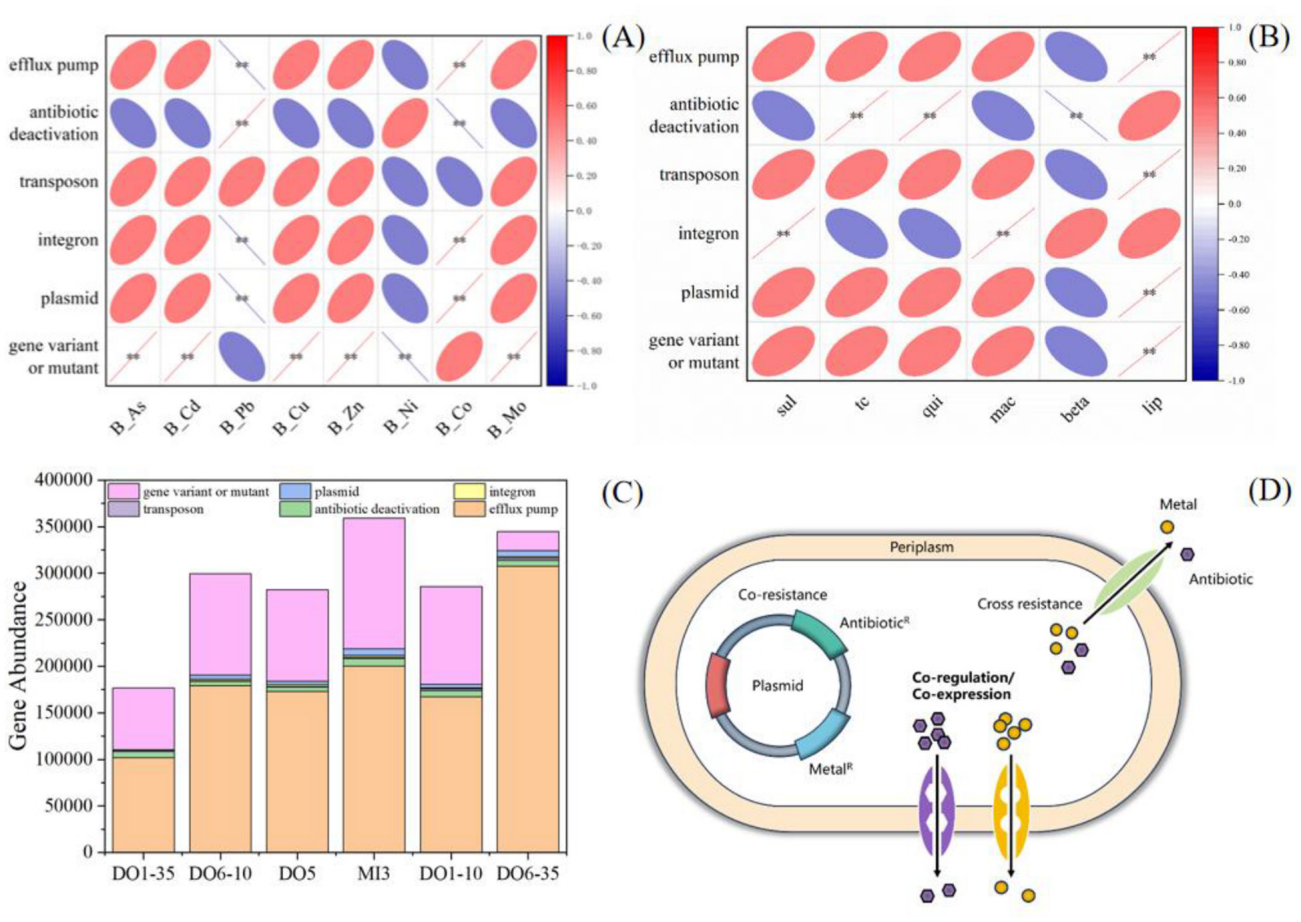
The effects of heavy metals and antibiotics on the MGEs. **(A, B)** Correlation of MGEs with heavy metals and antibiotics, **(C)** MGEs and Resistance Mechanisms Classification, **(D)** Mechanisms of cross-resistance of metal and antibiotic resistance

Co-selection of bacteria for antibiotic and metal resistance is important mainly because it maintains and promotes antibiotic resistance in bacterial populations in the absence of antibiotics. Co-selection is mainly caused by cross or co-resistance mechanisms; cross-resistance is a mechanism by which bacteria share a common tolerance to antibiotics and heavy metals in the same system, such as an efflux pump system (Chapman, 2003). Co-resistance refers to the carrying of both heavy metal resistance genes and antibiotic resistance genes on a single genetic element, such as a plasmid or transposon, or the presence of resistance genes in the same strain, where each resistance gene provides resistance to a different compound (Figure 4D) (Baker-Austin et al., 2006; Pal et al., 2015). MGEs such as plasmids, integrons, and transposons play an important role in resistance co-option (Frost et al., 2005).

Two surface sediment samples and four profile samples were selected for macro-genome sequencing, and the sequencing results were comparatively annotated to categorize the mobile MGEs, which were plasmid-related genes, transposons, integrons, efflux pumps, antibiotic deactivation and gene variant or mutant (Figure 4C). Studies have shown that the resistance mechanisms of microorganisms in this region are mainly composed of efflux pumps and gene mutations, and that an increase in antibiotic content directly leads to an increase in the number of efflux pumps (Figure 4B), as shown in samples DO6-35. Correlation analysis of the various types of resistance mechanisms with the content of heavy metals revealed that there is a significant positive correlation between As, Cd, Cu, Zn, and Mo and the mutation of resistance genes, suggesting that the presence of these metals will induce the mutations in cells leading to resistance (Figure 4A).

**Figure 5:**
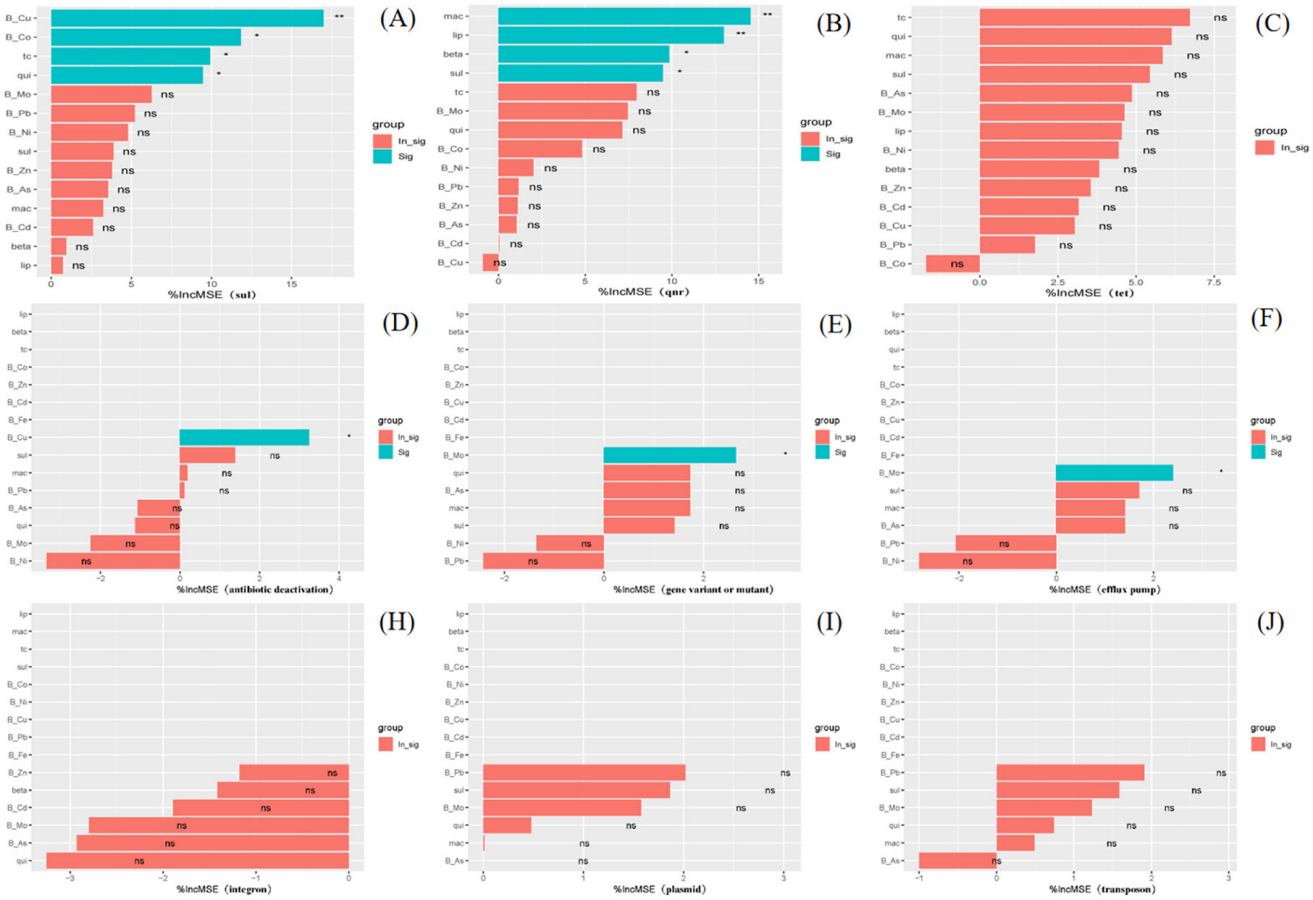
The contributions of heavy metals and antibiotics on the abundance of ARGs and MEGs with the Random Forest Analysis. **(A-C)** was referred to the ARGs, such as *sul, qnr* and *tet* genes, respectively. **(D-J)** was referred to the MGEs, such as antibiotic deactivation, gene variant or mutant, efflux pump, integron, plasmid and transposon.

Random forest analysis was carried out and it was found that all heavy metals contributed more to MGEs than antibiotics and that heavy metals have an important role in the inheritance of antibiotic resistance (Figures 5D–J). Co and Pb were significantly correlated with plasmids, integrons, and exocytosis pumps (Figure 4A), which may be due to the presence of a cooption mechanism. As it has been demonstrated that, in *Pseudomon*as aeruginosa, CzcRS controls the expression of the czcCBA exocytosis system that conferring resistance to Cd, Zn, and Co and decreasing the expression of OprD leading to increased resistance to carbapenems (Perron et al., 2004). It has also been reported that the multi-drug resistance efflux pump of Gram-positive Listeria *monocytogenes* excretes toxic metals (zinc, cobalt, and cadmium) in addition to the clindamycin, erythromycin, and cosamycin (Imran et al., 2019). Exposure to multiple metals is more favorable for the co-selection of antibiotic resistance than exposure to a single metal (Guo et al., 2014). Consequently, more research should also be conducted to develop more advanced detection and treatment measures (such as controlling heavy metals such as As, Cd, Cu, Zn, and Mo) to ensure water quality and soil health in offshore watersheds.

Furthermore, the sequential extraction analysis allowed us to differentiate the roles of specific heavy metal fractions. The weak acid-extractable (bioavailable) fraction showed the strongest positive correlations with ARGs (*sul1, sul2*) (Figures 5A–C) and key MGEs (e.g., plasmids and integrons) (Figures 5D–J), identifying it as the primary active driver for co-selection. This is because these readily available metals impose direct selective pressure, enriching bacteria carrying MGEs that harbor both metal and antibiotic resistance genes. In contrast, the residual fraction exhibited negligible correlations, confirming its inert role. The reducible and oxidizable fractions showed intermediate effects, representing a potential secondary reservoir. Thus, the bioavailability, not merely the total content, of heavy metals is the critical factor determining their impact on ARG propagation in sediments.

Advancement beyond previous studies: While the co-selection effect of heavy metals and antibiotics on ARGs is well-documented, this study provides a significant advance by moving beyond total metal concentrations. Our key novel finding is that the bioavailable fraction (weak acid-extractable) of heavy metals, not the total content, is the primary and most active driver of ARG proliferation in complex estuarine sediments. Furthermore, we quantitatively demonstrate that for critical ARGs like sul1 and sul2, the selective pressure exerted by these bioavailable metals can rival or even exceed that of co-occurring antibiotics (as shown by the random forest model where heavy metal contribution reached up to 72.6% for sul genes). This shifts the paradigm from monitoring total pollutants to assessing bioavailable and reactive fractions for accurate risk prediction. No prior study in similar estuarine systems has provided this level of mechanistic granularity linking metal speciation directly to ARG dissemination outcomes.

## 4 Conclusion

This study investigated the complex relationships among antibiotic resistance genes (ARGs), antibiotics, and heavy metals in sediment samples. The results indicated that tetracycline and sulfonamide antibiotics were the most prevalent antibiotic contaminants, while iron, zinc, and arsenic were the dominant heavy metals, highlighting the need for enhanced monitoring and management of these pollutants. Although antibiotics exhibited a greater overall influence on ARG abundance than heavy metals, certain heavy metals (Cu, Zn, Ni, Pb) still showed significant positive correlations with ARGs. This study analysis further revealed differential driving factors for distinct ARG types: for *tet*, the contribution of heavy metals was 50.47%, and that of antibiotics was 49.53%; for *sul* genes, the contribution of heavy metals was 72.62%, and that of antibiotic contribution was 27.38%; for *qnr* genes, heavy metal contribution was 20.67% and antibiotic contribution was 79.33%. It indicates that heavy metals also affect the distribution of ARGs. This study has certain limitations, including the absence of controlled experiments to mechanistically verify how heavy metals drive ARG spread, and insufficient analysis of long-term ecological risks under combined pollutant exposure. Future work should clarify the co-selection mechanisms between antibiotics and heavy metals in real environments. From a management perspective, we recommend implementing integrated monitoring of both contaminant types in sediments and incorporating them into ecological risk assessments to support estuary protection strategies.

## Data Availability

The raw sequence data reported in this paper have been deposited in the Genome Sequence Archive (GSA) in the National Genomics Data Center (NGDC) under accession number CRA040353.
