# Supplementary material for: Co-contamination of metal (loid)s and antibiotics induced the enrichment of antibiotic resistance genes in the coastal industrial basin
Source: Front Microbiol. 2026 Apr 1;17:1777820. doi: 10.3389/fmicb.2026.1777820 (PMC13079610; doi:10.3389/fmicb.2026.1777820)
Supplement: Supplementary file 1 [file Supplementary_file_1.docx]

Supplementary Material

Co-contamination of metal(loid)s and antibiotics induced the enrichment of antibiotic resistance genes in the coastal industrial basin

Changpeng Sang^a,b, ＃^, Wenyue Chen^a, ＃^, Enrui Li^a^, Meihua Lian^c*^, Zhiheng Li ^d^, Xiaojun Li^b^

^a^Key Laboratory of Industrial Ecology and Environmental Engineering (Ministry of Education, China), School of Environmental Science and Technology, Dalian University of Technology, Dalian, 116024, China

^b^Key Laboratory of Pollution Ecology and Environmental Engineering, Institute of Applied Ecology, Chinese Academy of Sciences, Shenyang, Liaoning 110016, China

^c^Key Laboratory of Wastewater Treatment Technology of Liaoning Province, Shenyang LigongUniversity,Shenyang 110159, China.

^d^School of Environmental Science and Engineering, Zhejiang Gongshang University, Hangzhou, Zhejiang 310018, China.

^＃^Changpeng Sang and Wenyue Chen contributed equally to this article.

*** Correspondence:**

lymeihua1983@163.com (M. Lian)

# Supplementary Figures and Tables

## Supplementary Figures


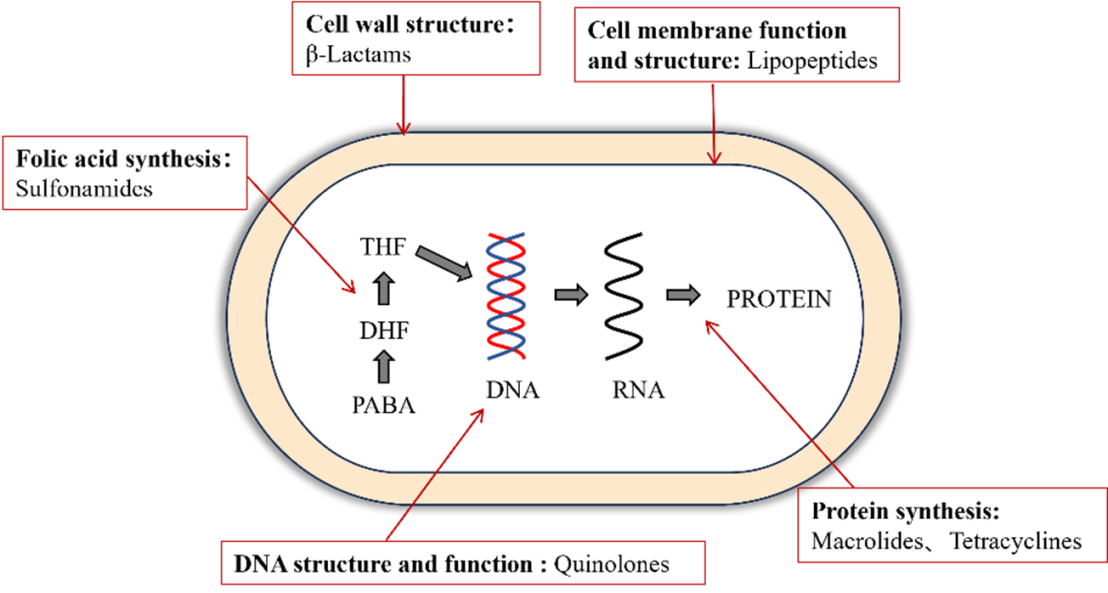


**Supplementary Figure 1.**Mechanisms of action of different antibiotics

## Supplementary Tables

**SupplementaryTable 1.**Antibiotic levels at each site(ng/g)

| Sampling Point | AMO | AMP | AZI | CIP | CTC | DTC | ENR | EYR | NOR | OFL | OTC | ROX | SDZ | SMR | SMT | SMZ | TC |
| --- | --- | --- | --- | --- | --- | --- | --- | --- | --- | --- | --- | --- | --- | --- | --- | --- | --- |
| UP1 | 6.95 | 0.96 | 0.40 | 0.37 | 3.74 | 26.01 | 0.64 | 4.74 | 3.63 | 2.17 | 20.49 | 2.84 | 43.11 | 5.33 | 3.55 | 2.85 | 35.20 |
| UP2 | 5.85 | 0.81 | 0.30 | 0.25 | 2.36 | 1.80 | 0.64 | 3.31 | 0.20 | 5.97 | 51.40 | 0.20 | 28.26 | 2.83 | 0.21 | 5.90 | 2.16 |
| UP3 | 6.44 | 0.82 | 0.31 | 0.29 | 2.94 | 73.19 | 0.71 | 3.11 | 13.76 | 2.57 | 23.15 | 7.84 | 27.84 | 3.57 | 10.48 | 2.32 | 114.84 |
| MI1 | 6.29 | 0.55 | 0.35 | 0.28 | 2.78 | 5.68 | 0.58 | 3.01 | 0.68 | 5.26 | 49.68 | 0.54 | 26.09 | 2.27 | 0.57 | 5.14 | 6.44 |
| MI2 | 1.84 | 0.19 | 0.32 | 0.22 | 2.36 | 101.44 | 0.20 | 3.05 | 16.25 | 4.75 | 40.95 | 9.68 | 28.87 | 2.46 | 17.80 | 5.47 | 135.73 |
| MI3 | 14.98 | 1.69 | 0.43 | 0.34 | 3.67 | 299.84 | 1.77 | 3.79 | 32.12 | 8.11 | 80.25 | 27.38 | 33.72 | 3.93 | 44.81 | 7.94 | 325.07 |
| DO1 | 5.98 | 0.60 | 0.61 | 0.52 | 4.65 | 16.28 | 0.62 | 4.87 | 2.29 | 5.07 | 53.25 | 1.52 | 44.68 | 5.01 | 1.73 | 7.21 | 19.32 |
| DO2 | 3.99 | 0.50 | 0.23 | 0.17 | 1.61 | 58.87 | 0.43 | 2.22 | 7.15 | 7.43 | 65.19 | 5.38 | 19.70 | 2.72 | 6.24 | 8.92 | 69.37 |
| DO3 | 3.00 | 0.35 | 0.2 | 0.15 | 1.51 | 29.09 | 0.29 | 1.91 | 4.66 | 4.20 | 42.50 | 2.98 | 19.11 | 2.25 | 5.08 | 4.51 | 0 |
| DO4 | 2.25 | 0.23 | 0 | 0 | 0.62 | 17.61 | 0.25 | 0.77 | 2.60 | 4.79 | 51.49 | 1.91 | 6.58 | 0.80 | 2.89 | 4.49 | 22.43 |
| DO5 | 1.95 | 0.26 | 0 | 0 | 0.63 | 11.86 | 0.21 | 0.57 | 1.47 | 3.50 | 30.35 | 1.32 | 5.53 | 0.75 | 1.82 | 2.93 | 16.08 |
| DO6 | 1.99 | 0.23 | 0.33 | 0.28 | 2.89 | 7.57 | 0.20 | 1.42 | 1.32 | 3.12 | 32.34 | 0.73 | 12.53 | 1.40 | 1.57 | 3.55 | 11.75 |
| DO7 | 0.63 | 0 | 0.18 | 0.21 | 2.15 | 8.77 | 0 | 1.50 | 1.44 | 9.48 | 84.18 | 1.00 | 12.89 | 1.74 | 1.65 | 11.34 | 15.42 |
| DO1-5 | 1.51 | 0.14 | 0.28 | 0.26 | 2.24 | 7.68 | 0.16 | 2.38 | 0.90 | 6.53 | 59.81 | 0.70 | 22.11 | 1.92 | 0.82 | 5.50 | 9.36 |
| DO1-10 | 0.72 | 0 | 0.49 | 0.53 | 4.94 | 8.22 | 0 | 1.02 | 0.97 | 1.27 | 11.15 | 0.96 | 8.96 | 1.03 | 1.26 | 1.29 | 10.11 |
| DO1-15 | 0.15 | 0 | 0.48 | 0.36 | 3.73 | 0. | 0 | 0.78 | 0.58 | 1.10 | 11.24 | 0 | 7.43 | 0.89 | 0.58 | 1.53 | 5.29 |
| DO1-20 | 0.17 | 0 | 0.50 | 0.40 | 3.85 | 0.26 | 0 | 0.97 | 0.28 | 0.46 | 4.71 | 0 | 8.60 | 1.11 | 0.26 | 0.40 | 2.79 |
| DO1-25 | 0.11 | 0 | 0.19 | 0.22 | 2.26 | 0 | 0 | 0.21 | 0.38 | 0.35 | 2.93 | 0.28 | 2.23 | 0.18 | 0.34 | 0.33 | 3.21 |
| DO1-30 | 0.15 | 0 | 0.57 | 0.13 | 0.74 | 0 | 0 | 0.60 | 0.40 | 0.10 | 0.47 | 0.35 | 5.40 | 0.57 | 0.35 | 1.27 | 3.56 |
| DO1-35 | 0 | 0 | 0.44 | 0 | 0.10 | 0 | 0 | 0.48 | 0.20 | 0 | 0 | 0.22 | 4.10 | 0.19 | 0.27 | 0.29 | 1.08 |
| DO1-40 | 0 | 0 | 0.1 | 0 | 0 | 0 | 0 | 0.57 | 0 | 0 | 0 | 0.18 | 1.02 | 0 | 0.48 | 0 | 0.72 |
| DO1-45 | 0 | 0 | 0 | 0 | 0 | 0 | 0 | 0 | 0 | 0 | 0 | 0 | 0 | 0 | 0 | 0 | 0 |
| DO1-50 | 0 | 0 | 0 | 0 | 0 | 0 | 0 | 0 | 0 | 0 | 0 | 0 | 0 | 0 | 0 | 0 | 0 |
| DO1-55 | 0 | 0 | 0 | 0 | 0 | 0 | 0 | 0 | 0 | 0 | 0 | 0 | 0 | 0 | 0 | 0 | 0 |
| DO1-60 | 0 | 0 | 0 | 0 | 0 | 0 | 0 | 0 | 0 | 0 | 0 | 0 | 0 | 0 | 0 | 0 | 0 |
| DO6-5 | 18.02 | 0.77 | 0.40 | 1.65 | 13.91 | 102.61 | 1.85 | 28.37 | 21.12 | 0.19 | 1.78 | 11.07 | 246.39 | 4.83 | 2.38 | 5.92 | 192.64 |
| DO6-10 | 8.99 | 0.63 | 0.43 | 0.75 | 7.13 | 60.22 | 0.94 | 21.38 | 1.07 | 0 | 0.31 | 6.76 | 187.36 | 5.24 | 3.40 | 2.41 | 9.47 |
| DO6-15 | 5.75 | 0.65 | 0.29 | 0.82 | 6.87 | 39.47 | 0.58 | 13.43 | 1.83 | 0.13 | 1.28 | 4.68 | 117.71 | 3.41 | 0.19 | 5.19 | 16.19 |
| DO6-20 | 1.99 | 0.57 | 0.30 | 0.30 | 2.93 | 14.52 | 0.22 | 5.61 | 1.06 | 0 | 0.60 | 1.70 | 48.18 | 2.59 | 13.29 | 3.21 | 10.61 |
| DO6-25 | 1.29 | 0.71 | 0.27 | 0.46 | 4.92 | 9.91 | 0.13 | 2.68 | 1.74 | 0 | 0.49 | 1.01 | 23.72 | 2.23 | 0.65 | 5.10 | 18.25 |
| DO6-30 | 1.63 | 0.25 | 0.22 | 0.16 | 4.80 | 8.87 | 0.17 | 0.86 | 0.81 | 0.11 | 0.92 | 0.87 | 7.73 | 3.38 | 0.93 | 4.00 | 7.21 |
| DO6-35 | 0.75 | 1.53 | 0.43 | 0 | 0.85 | 2.23 | 0.14 | 0.46 | 0.29 | 0 | 0.74 | 0.26 | 4.40 | 1.07 | 0.75 | 2.64 | 2.88 |
| DO6-40 | 0.14 | 0.73 | 0.15 | 0 | 0.14 | 1.67 | 0.13 | 0 | 0 | 0 | 0 | 0.19 | 0.54 | 0.18 | 0.10 | 0.61 | 0.56 |
| DO6-45 | 0 | 0 | 0 | 0 | 0 | 0.33 | 0 | 0 | 0 | 0 | 0 | 0 | 0.14 | 0 | 0 | 0 | 0 |
| DO6-50 | 0 | 0 | 0 | 0 | 0 | 0 | 0 | 0 | 0 | 0 | 0 | 0 | 0 | 0 | 0 | 0 | 0 |
| DO6-55 | 0 | 0 | 0 | 0 | 0 | 0 | 0 | 0 | 0 | 0 | 0 | 0 | 0 | 0 | 0 | 0 | 0 |
| DO6-60 | 0 | 0 | 0 | 0 | 0 | 0 | 0 | 0 | 0 | 0 | 0 | 0 | 0 | 0 | 0 | 0 | 0 |

**SupplementaryTable 2.**Heavy metal(loid)s levels at each site(mg/kg)

| Sampling Point | As | Cd | Pb | Cu | Zn | Ni | Co | Mo |
| --- | --- | --- | --- | --- | --- | --- | --- | --- |
| UP1 | 8.96 | 1.07 | 40.76 | 9.61 | 155.22 | 21.09 | 5.56 | 0.44 |
| UP2 | 4.39 | 0.15 | 0.42 | 47.34 | 216.23 | 15.25 | 4.08 | 0.12 |
| UP3 | 4.08 | 0.91 | 84.16 | 41.74 | 292.16 | 26.60 | 3.72 | 0.40 |
| MI1 | 92.18 | 239.39 | 66.64 | 111.96 | 4563.64 | 21.53 | 10.82 | 0.40 |
| MI2 | 175.86 | 5.28 | 70.66 | 81.85 | 1223.98 | 15.20 | 5.92 | 0.28 |
| MI3 | 110.92 | 6.34 | 21.99 | 9.58 | 413.11 | 12.64 | 7.72 | 0.84 |
| DO1 | 637.53 | 51.32 | 278.24 | 603.54 | 5323.25 | 17.37 | 11.71 | 0.20 |
| DO2 | 821.67 | 43.16 | 3..026 | 546.76 | 6158.34 | 15.04 | 12.12 | 0.81 |
| DO3 | 55.70 | 15.04 | 214.34 | 194.17 | 2908.43 | 6.41 | 8.41 | 0.17 |
| DO4 | 226.90 | 8.39 | 187.87 | 155.52 | 2291.26 | 5.69 | 3.78 | 0.09 |
| DO5 | 133.90 | 9.65 | 176390 | 171.80 | 2415.90 | 7.39 | 9.37 | 0.15 |
| DO6 | 19.69 | 4.97 | 90.72 | 38.33 | 703.31 | 4.26 | 5.39 | 0.03 |
| DO7 | 38.25 | 10.93 | 143.99 | 92.03 | 1749.18 | 5.97 | 6.95 | 0.04 |
| DO1-5 | 735.36 | 51.87 | 329.09 | 638.03 | 5890.09 | 27.12 | 12.70 | 0.48 |
| DO1-10 | 101.66 | 82.95 | 194.64 | 783.25 | 5540.14 | 21.91 | 12.97 | 4.12 |
| DO1-15 | 807.56 | 47.64 | 289.25 | 521.67 | 4644.10 | 22.23 | 10.31 | 1.46 |
| DO1-20 | 226.81 | 17.81 | 240.73 | 180.63 | 2089.89 | 15.41 | 8.71 | 0.59 |
| DO1-25 | 21.43 | 2.95 | 52.85 | 28.95 | 293.66 | 4.25 | 4.45 | 0.34 |
| DO1-30 | 43.03 | 5.02 | 75.80 | 59.13 | 477.62 | 4.24 | 4.28 | 0.32 |
| DO1-35 | 27.58 | 3.31 | 46.85 | 39.30 | 299.37 | 4.16 | 4.12 | 0.23 |
| DO1-40 | 18.60 | 2.09 | 33.61 | 29.00 | 216.40 | 4.03 | 3.73 | 0.25 |
| DO1-45 | 5.29 | 0.21 | 14.33 | 8.62 | 42.01 | 3.49 | 3.42 | 0.11 |
| DO1-50 | 0.44 | 0.03 | 4.85 | 2.87 | 5.24 | 3.38 | 3.99 | 0.10 |
| DO1-55 | 0.26 | 0.02 | 4.25 | 2.07 | 4.34 | 2.82 | 3.31 | 0.12 |
| DO1-60 | 0.22 | 0.01 | 2.06 | 1.44 | 4.25 | 3.54 | 4.11 | 0.08 |
| DO6-5 | 217.15 | 38.06 | 606.78 | 209.34 | 4585.48 | 16.96 | 21.16 | 3.86 |
| DO6-10 | 305.10 | 66.26 | 408.30 | 314.31 | 5470.22 | 16.71 | 26.63 | 10.90 |
| DO6-15 | 391.99 | 27.55 | 429.91 | 382.29 | 4692.68 | 18.45 | 18.86 | 4.99 |
| DO6-20 | 804.56 | 65.74 | 479.32 | 397.49 | 6906.58 | 19.39 | 20.33 | 7.80 |
| DO6-25 | 165.41 | 9.48 | 57.18 | 4.28 | 1448.23 | 6.21 | 1.45 | 2.39 |
| DO6-30 | 173.63 | 9.53 | 59.15 | 4.28 | 1456.42 | 6.32 | 1.40 | 1.93 |
| DO6-35 | 272.40 | 14.42 | 56.16 | 2.75 | 2541.88 | 4.38 | 1.18 | 0.61 |
| DO6-40 | 201.57 | 7.67 | 57.01 | 3.49 | 1620.02 | 4.37 | 1.47 | 0.63 |
| DO6-45 | 315.24 | 13.82 | 71.04 | 3.93 | 1712.34 | 5.54 | 1.36 | 1.49 |
| DO6-50 | 2.32 | 1.42 | 25.58 | 5.03 | 116.65 | 3.63 | 3.86 | 0.14 |
| DO6-55 | 1.75 | 0.46 | 15.09 | 4.13 | 61.66 | 3.63 | 3.86 | 0.09 |
| DO6-60 | 1.22 | 0.35 | 1025 | 2.29 | 26.36 | 2.05 | 2.25 | 0.05 |

**SupplementaryTable 3.Relative abundance of ARGs at each site(%)**

| Sampling Point | *tetA* | *tetM* | *sul1* | *sul2* | *qnrS* | *qnrA* |
| --- | --- | --- | --- | --- | --- | --- |
| UP1 | 0.0064 | 0.0383 | 0.0307 | 0.2119 | 0.0003 | 0.0002 |
| UP2 | 0.0054 | 0.0308 | 0.0023 | 0.5261 | 0.0003 | 1.7200E-05 |
| UP3 | 0.0057 | 0.0297 | 0.0967 | 0.2190 | 0.0002 | 0.0007 |
| MI1 | 0.0054 | 0.0237 | 0.0071 | 0.4422 | 0.0002 | 5.5606E-05 |
| MI2 | 0.0017 | 0.0243 | 0.1219 | 0.4337 | 0.0002 | 0.0010 |
| MI3 | 0.0133 | 0.0345 | 0.3545 | 0.8250 | 0.0003 | 0.0029 |
| DO1 | 0.0061 | 0.0442 | 0.0208 | 0.4785 | 0.0005 | 0.0002 |
| DO2 | 0.0034 | 0.0168 | 0.0751 | 0.6913 | 0.0002 | 0.0006 |
| DO3 | 0.0029 | 0.0175 | 0.0350 | 0.4392 | 0.0001 | 0.0003 |
| DO4 | 0.0023 | 0.0066 | 0.0210 | 0.4830 | 6.4502E-05 | 0.0002 |
| DO5 | 0.0021 | 0.0051 | 0.0136 | 0.2734 | 5.6061E-05 | 0.0001 |
| DO6 | 0.0019 | 0.0119 | 0.0113 | 0.3225 | 0.0003 | 7.5762E-05 |
| DO7 | 0.0006 | 0.0109 | 0.0135 | 0.7727 | 0.0002 | 8.9256E-05 |
| DO1-5 | 0.0014 | 0.0206 | 0.0091 | 0.5680 | 0.0002 | 6.9033E-05 |
| DO1-10 | 0.0007 | 0.0634 | 0.0098 | 0.5017 | 0.0005 | 7.2985E-05 |
| DO1-15 | 0.0001 | 0.0302 | 0.0051 | 0.4965 | 0.0003 | 4.3874E-06 |
| DO1-20 | 0.0002 | 0.03360 | 0.0028 | 0.4870 | 0.0003 | 2.3575E-06 |
| DO1-25 | 0.0001 | 0.0203 | 0.0031 | 0.4569 | 0.0002 | 2.4402E-05 |
| DO1-30 | 0.0001 | 0.0595 | 0.0038 | 0.4435 | 0.0006 | 3.1973E-05 |
| DO1-35 | 0.0001 | 0.02826 | 0.0020 | 0.4502 | 0.0002 | 1.3615E-05 |
| DO1-40 | 0.0001 | 0.0722 | 0.0020 | 0.4848 | 0.0007 | 1.6857E-05 |
| DO1-45 | 0.0001 | 0.0442 | 0.0016 | 0.3437 | 0.0004 | 1.3789E-05 |
| DO1-50 | 0.0001 | 0.0471 | 0.0018 | 0.3301 | 0.0004 | 1.3397E-05 |
| DO1-55 | 0.0002 | 0.0531 | 0.0018 | 0.2539 | 0.0006 | 1.2123E-05 |
| DO1-60 | 6.9919E-05 | 0.0394 | 0.0015 | 0.2259 | 3.5165E-05 | 1.2363E-05 |
| DO6-5 | 0.0157 | 0.2574 | 0.2023 | 0.0167 | 0.0014 | 0.0009 |
| DO6-10 | 0.0076 | 0.1729 | 0.0086 | 0.0031 | 0.0007 | 0.0005 |
| DO6-15 | 0.0059 | 0.0185 | 0.0014 | 0.0013 | 0.0006 | 0.0004 |
| DO6-20 | 0.0020 | 0.04859 | 0.03818 | 5.7383E-05 | 0.0003 | 0.0002 |
| DO6-25 | 0.0011 | 0.0234 | 0.0184 | 4.9970E-05 | 0.0005 | 0.0003 |
| DO6-30 | 0.0014 | 0.0081 | 0.0064 | 9.2657E-05 | 0.0005 | 0.0004 |
| DO6-35 | 0.0011 | 0.0039 | 0.0031 | 7.8238E-05 | 0.0006 | 0.0004 |
| DO6-40 | 0.0013 | 0.8432 | 0.0026 | 7.3843E-05 | 0.0002 | 0.0001 |
| DO6-45 | 0.0002 | 0.0066 | 0.0057 | 1.0379E-05 | 9.1443E-05 | 6.2767E-05 |
| DO6-50 | 0.0001 | 0.0067 | 0.0057 | 4.3619E-05 | 7.9698E-05 | 5.4704E-05 |
| DO6-55 | 0.0002 | 0.0066 | 0.0016 | 1.9269E-05 | 3.0237E-05 | 2.0755E-05 |
| DO6-60 | 7.7265E-05 | 0.0060 | 0.0068 | 1.3527E-05 | 1.6712E-05 | 1.1471E-05 |

**SupplementaryTable 4. Random Forest Analysis**

| %MSE | plasmid | integron | transposon | efflux pump | antibiotic deactivation | gene variant or mutant | *tet* | *sul* | *qnr* |
| --- | --- | --- | --- | --- | --- | --- | --- | --- | --- |
| Heavy metal | 60.43 | 65.31 | 66.54 | 73.63 | 78.75 | 62.57 | 50.47 | 72.62 | 20.67 |
| Antibiotic | 39.57 | 34.69 | 33.46 | 26.37 | 21.25 | 37.43 | 49.53 | 27.38 | 79.33 |

**SupplementaryTable 5. MGEs and resistance mechanisms（num. of reads）**

| %MSE | plasmid | integron | transposon | efflux pump | antibiotic deactivation | gene variant or mutant |
| --- | --- | --- | --- | --- | --- | --- |
| DO1-35 | 1348 | 272 | 304 | 102028 | 6590 | 66186 |
| DO6-10 | 4384 | 1772 | 786 | 179188 | 4622 | 108906 |
| DO5 | 3886 | 1558 | 514 | 173203 | 4790 | 98190 |
| MI3 | 7418 | 1608 | 1496 | 200166 | 8252 | 139988 |
| DO1-10 | 3524 | 1202 | 1432 | 167186 | 7050 | 105100 |
| DO6-35 | 6694 | 1296 | 2838 | 307722 | 5820 | 20354 |
